# Traditional sports and games among the Sámi people in Northern Fennoscandia (Sápmi): an ethnobiological perspective

**DOI:** 10.1186/s13002-022-00517-9

**Published:** 2022-03-19

**Authors:** Isak Lidström, Ingvar Svanberg, Sabira Ståhlberg

**Affiliations:** 1 Department of History, Stockholm University, Historia, 106 91 Stockholm, Sweden; 2 Institute for Russian and Eurasian Studies, Uppsala University, Box 514, 751 20 Uppsala, Sweden

**Keywords:** Environmentalization, Environmental sustainability, Fish glue, Indigenous environmental knowledge, Plant materials, Reindeer, Sports history, Traditional games

## Abstract

**Introduction:**

Modern sports equipment is nowadays manufactured industrially according to globally accepted and standardized models, but traditionally tools for play and games were prepared from materials found in the local environment. The objective of this article is to investigate various aspects of Sámi local knowledge about organisms used for their material culture of traditional sports and games in northern Fennoscandia (Sápmi). What functions did the surrounding biota have in the production of equipment used in sports and games?

**Methods:**

A qualitative method was used; the ethnographic literature and travel narratives have been analyzed particularly for descriptions and notes on traditional games, toys, and sports.

**Results:**

Before the turn of the twentieth century, bats, balls, and skis were seldom produced in factories, but by children and adults who utilized available materials from the surrounding environment. The manufacture of tools for play and games was characterized by a rich creativity in the use of various biological and natural resources. A wide range of such resources is presented in this article, among them the bracket fungus *Fomitopsis betulina,* used for making balls, reindeer antlers utilized for lassoing contests, and pine bark painted with reindeer blood, prepared for playing cards. We also highlight how tools usually associated with means of transport could switch functions and serve playful and competitive purposes, such as skis made of compression pine or walking sticks of birch: The former were used in skiing races, and the latter appeared in fencing competitions.

**Conclusion:**

The industrialization of the material culture of sports has been contributed to a loss of local knowledge and familiarity with locally available organic stuffs for producing equipment for play and games. By reconnecting with previous knowledge of traditional games, we discover a potentially new direction for modern sports and games, shifting from globalization to environmentalization. Such an environmentalization could permit the local environmental context define the content, meaning and structure of sports, and simultaneously enrich both sports and outdoor life.

## Introduction

Traditionally, sports and games have been performed with the help of locally available materials from the surrounding landscape [1–4]. This use presupposes a broad material knowledge of the qualities, usefulness, and properties of various organic (algae, fungi, plants, animals) and nonliving sources such as snow, ice, stones, or water, in the ecosystem. Local knowledge about resources in the landscape was deeply embedded in the culture, lifestyle, perceptions, world view, and thinking, and also often in the toponyms used by the local inhabitants; it is therefore a topic of great interest for ethnobiologists [5, 6].

**Fig. 1:**
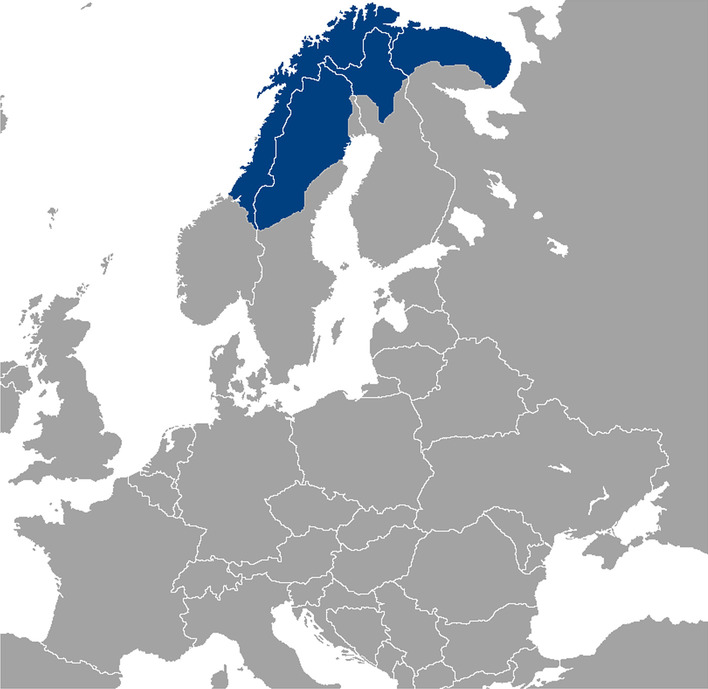
The area of Sápmi, where different Sámi groups live (Map Wikimedia Commons)

Indigenous nomadic and sedentary Sámi (also spelled Saami) form several groups (Fig. 1) in the far north of Fennoscandia. This area in Northern Europe, extending over the borders of Norway, Sweden, Finland, and Russia, is also known as *Sápmi*. The Sámi groups possessed earlier a wide range of local knowledge and practices, which were reflected in every aspect of their cultures, languages, and ways of living [7].

A historical example was recorded in the seventeenth century, showing that the Sámi were very committed to sports and games: “they are good at running; they jump over streams that are about three fathoms wide [5.38 m],” wrote the student Nicolaus Lundius (1656–1726) in 1674. He was a Sámi himself from Nasa Mountain (Lule Sámi *Násávárre*), studying at Uppsala University to become a vicar. Lundius praised in his manuscript also their swimming skills in cold lakes and streams. Sámi men jumped over brooks with the support of poles, and the women were just as expert at these physical exercises as the men [8]. A later example tells that when Sámi children in Arvidsjaur (Pite Sámi *Árvehávvre*) played a local kind of pick-up-pins (Ume Sámi *schärkot*), an internationally known game requiring physical skill, they used a bundle of wooden sticks, a small stone, and a hide of reindeer, on which they played [9].

**Fig. 2:**
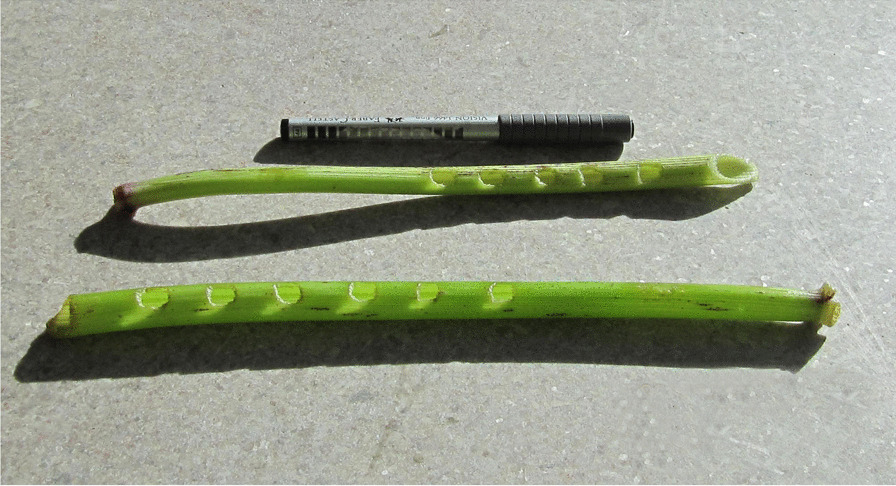
Two Sámi *fádnu* flutes with six finger holes made from the angelica plant (Photo Leif Wäppling 2010. Wikimedia Commons. CC BY-SA 3.0)

A further example is the use of stems of a culturally significant plant, *Angelica archangelica* L. It has traditionally been used for making all kinds of things, smoking pipes, toys, and a simple flute, *fádnu* (North Sámi) or *sjurggá* (Ume Sámi) (Fig. 2). An angelica flute seems to be a unique musical instrument worldwide; it is known only from the Sámi area [10]. Musical instruments were also created from the crop (sublingual pouch) of a ptarmigan (*Lagopus* sp*.*), or some other bird. When closing it at one end and letting out air through the other, it would produce a sound. Stems of goat willow, *Salix caprea* L., and bark from rowan, *Sorbus aucuparia* L., were also used to make small flutes. The Skolt Sámi used feathers of whooper swan, *Cygnus cygnus* (L.), for making a little pipe, with which they attracted hazel grouses, *Tetrastes bonasia* (L.) [11, 12].

Simple toys of ephemeral nature created by Sámi children were used for playing, for instance, animal farms. While peasant children used cones of Norwegian spruce, *Picea abies* (L.) H. Karst, and Scots pine, *Pinus sylvestris* L., to represent the livestock, mainly reindeer, Sámi children along the coast would use various kinds of mussel shells for the same animals [3]. Among these were seashells such as *Pecten maximus* (L.), known as *heasttaskálžu* “horse shell” in North Sámi, *Mytilus edulis* L., known as *bohkkaskálžu* “billy-goat shell” in North Sámi, and other bivalve species along the Norwegian Atlantic coast [13]. Children would also create necklaces out of the vertebrae from northern pike, *Esox lucius* L. A larger fish gas bladder could be pressed so that it bursts with a bang [11, 12]. Boys fished actively in the summer in rivers and lakes with their bare hands, a willow loop at the end of a stick, or with nets an arm long they had created themselves [12].

**Fig. 3:**
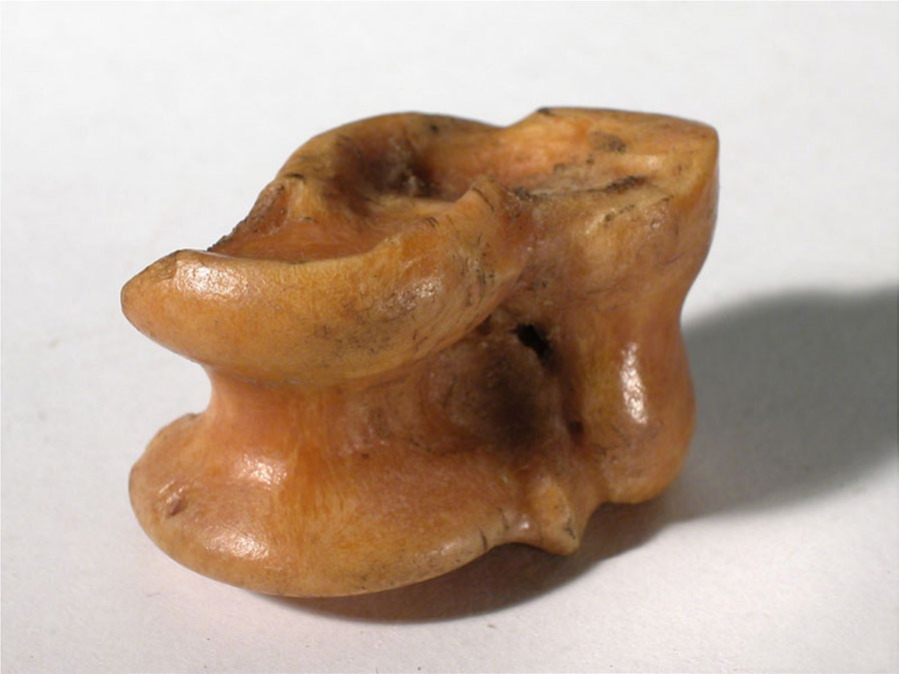
Kneecap of reindeer used by Sámi children in Finnish Petsamo as a toy reindeer (Courtesy of The Nordic Museum, Stockholm)

Wooden toys were very popular: small reindeer, sleighs, boats (also made of tree bark, preferably downy birch, *Betula pubescens* Ehrh., and other things of wood have been extensively documented in the Sámi material culture. Girls sewed beautiful clothes from textiles and reindeer skin for their wooden dolls. Dolls, however, seem to have been introduced later to the Sámi; they do not occur in the early travel accounts. Wooden pieces containing ant-made holes, sticks, branches, birch bark, reindeer bones, and strange stones could also become toys, often representing reindeer, cows, sheep, or horses; whole herds and trade caravans were created with them (Fig. 3). Several spinning tops and weather vanes (weathercocks) are recorded; some also made a sound when blown into by humans or by the wind [11, 12].

**Fig. 4:**
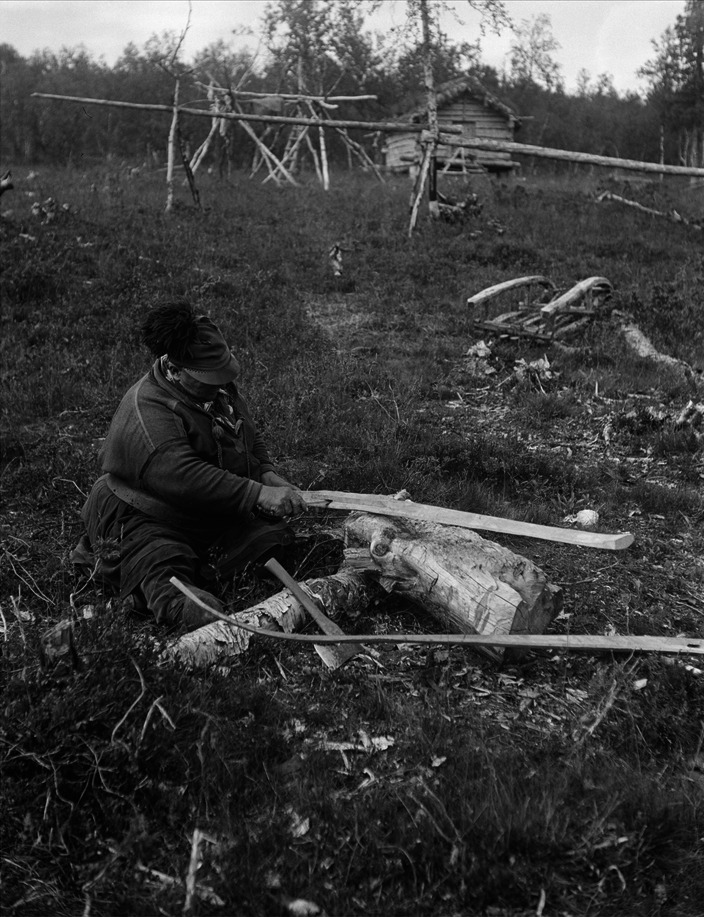
Nils Nilsson Skum (1872–1951), Sjiskavare, Girjas, Sweden, making skis (Photo Ernst Manker. Courtesy of The Nordic Museum, Stockholm)

In all these cases, the users or players themselves manufactured the equipment needed for the games or physical exercises. The tools and accessories of Sámi games and contests were made of locally available materials such as antlers, bones, grass, hides, and wood. The Sámi had previously a broad understanding and carefully used the available biological and natural resources surrounding them for their daily needs. This attentiveness was extended also to the sports and games. The traditional material culture of the games of the Sámi and other people of the north differ drastically from the material culture of sports prevalent today in the region [13] (Fig. 4). Modern sports equipment are manufactured industrially and according to globally accepted and standardized patterns and models. They draw on a multitude of natural resources for production and are often transported over long distances. These products have little connection with the local people or their environmental conditions. Regardless where in the world a tennis match is played, one can be sure that the ball is made of the same materials and has the same color and size as any other tennis ball. This circumstance is a consequence of the claims to universality of modern sports: all achievements and results must be measurable and comparable with each other on the global level [14].

Before the age of modernization (the transition from a premodern to a modern society), sports and games were not characterized by such universalism. The lack of standardization is reflected especially in the traditional material culture related to pastime activities. Before the turn of the twentieth century, for instance balls, bats, skates, skis, or boats were seldom produced in factories. The participants usually created the equipment themselves, employing the knowledge of the earlier generations and their own imagination and using locally available material from the environment. The increasing industrialization of the material culture of sports and games during the past century has been contributed to a loss of local knowledge and familiarity with locally available organic materials for producing equipment for play and games. A few Sámi children may possess such knowledge to some extent today, but traditional skills are of marginal importance in the modern lifestyle, and they are not widely distributed anymore [13].

Traditional games of the past are supposed to be more closely linked to the Dutch historian Johan Huizinga’s definition of play, stating that it is a practice separated from ordinary life. Play does not serve any material purpose, according to him; rather, it is performed for the sake of play itself [15]. In the case of the Sámi, and many other people closely connected with their environment, this is not completely true. Sámi children prepared for their future life through games and sports, and adults kept up their physical and mental fitness through sports especially during the long winter months. Children imitated grown-ups and exercised through play the necessary skills for their adult tasks, such as lassoing reindeer, driving sleighs, snaring birds, killing lemmings, *Lemmus lemmus* (L.), with a stick, and stretching their hides to dry, and so forth. As adults, they would use the same techniques for killing and preparing bigger animals [11].

**Fig. 5:**
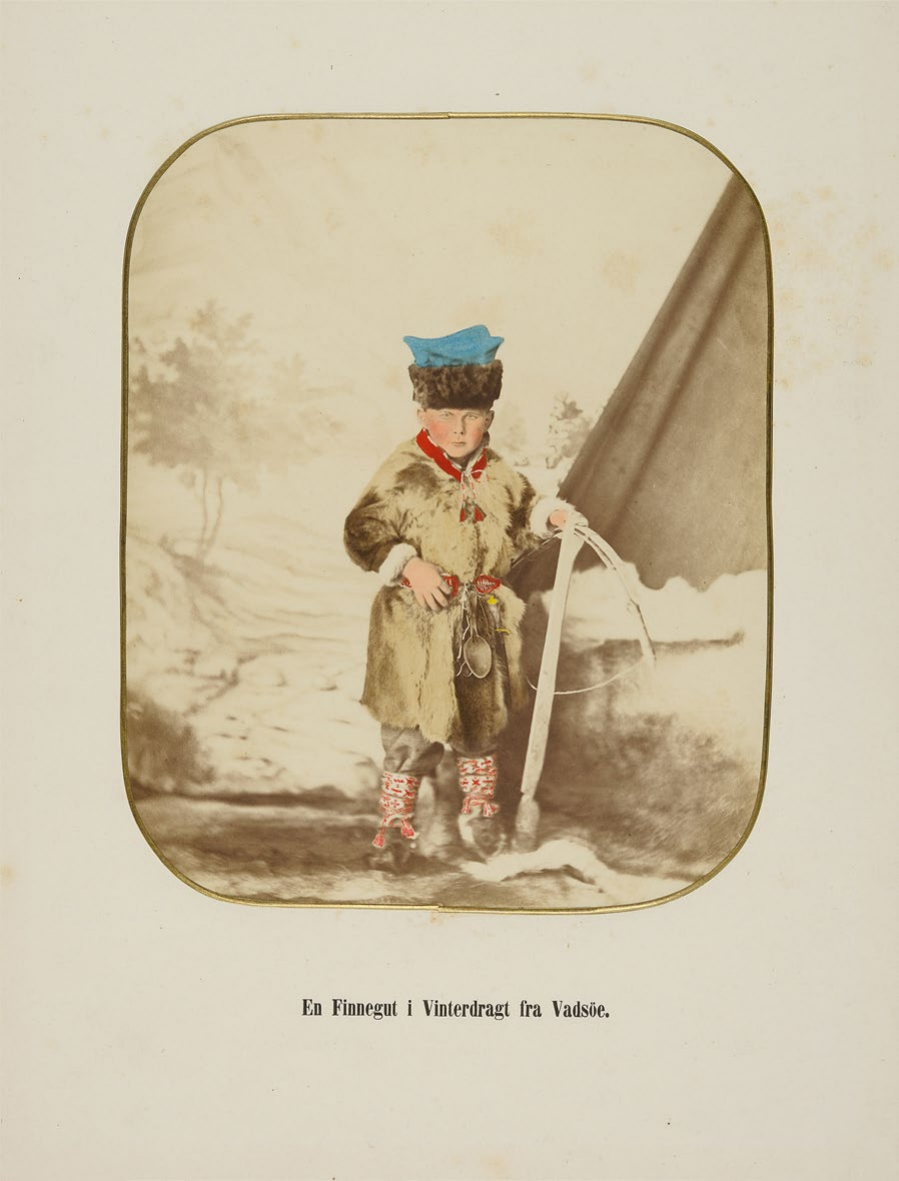
A Sámi boy in winter clothing in Vadsøe, Norway, carrying a wooden crossbow. This hand-colored photo is taken between 1857 and 1870 (Photo Marcus Selmber, Preus Museum, Norway. Creative Commons Attribution 2.0)

**Fig. 6:**
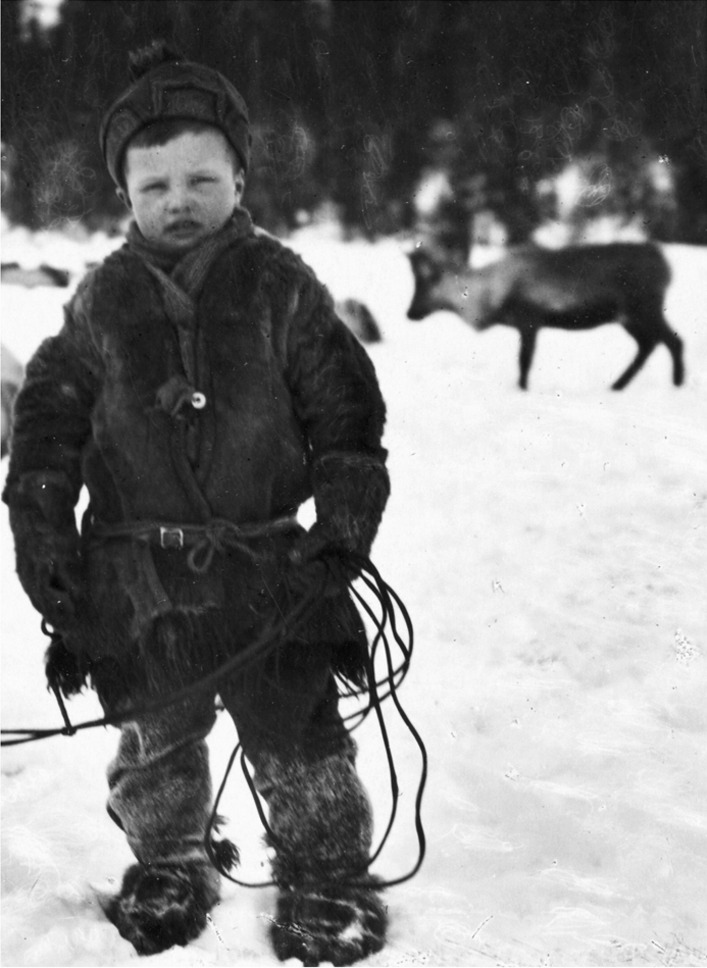
Sámi boy Klas Andersson from Frostviken, Strömsund, Jämtland, Sweden, with lasso in 1923. Unknown photographer. Wikimedia Commons, CC BY-SA 3.0

Playing was the way to learn from a very early age to deal with and manage environmental, economic, social, and other situations in a harsh climate (Figs. 5, 6]. When the children were old enough (from age 6–7 years) to perform “serious” tasks, they were well prepared and carried out the tasks with great enthusiasm and ease. They had already lassoed dogs and each other for several years, chased one another in a “reindeer chase,” or in the case of the coastal Sámi, competed with small boats, created “market” areas for “trade,” trained fishing, shooting with bow and arrow (among some groups, the children had to practice shooting and hit a target before getting food), etc. Children also played at building small huts out of branches, stones, or snow. The huts were big enough to sit in. They also played at choosing and finding firewood, preparing coffee and tea or cheese, cooking and consuming meat, and inviting guests (hospitality). Few games were divided according to gender in early childhood, yet only girls played with dolls, preparing small figures, clothes and other objects of simple materials. Older girls learned through games how to work with leather and fur, sewing and tin thread needlework, and also milking, while boys were trained in working with wood and using roots for basket weaving. Among the Sámi not only the children played, however; also the adults had their games and sports, which were more complex and challenging. The games contributed to better physical and mental skills, and to enriching their knowledge both of themselves and the environment. There were many different kinds of games; some were imported from neighboring people, while others were invented locally and remained group-specific. The Finnish scholar Toivo I. Itkonen divided Sámi games and playing into games with and without tools; games on ice and snow; social customs imitations (going to church or court, meeting a guest, wedding, etc.); ball, throwing, and arrow sports; using “technical” tools; strength and skills games; dice, board, and card games; entertainment and contests [11–13].

## Aim, cultural context, and methods

The Sámi are an indigenous population consisting of several groups, residing in an area reaching across the northern regions of Norway, Sweden, Finland, and the European part of Russia. The contemporary population is estimated to be between 75,000 and 100,000. The Sámi languages are a group of Uralic languages related to the Finnish languages [16].

Presenting some traditional Sámi leisure activities, we explore in this article how the material culture in the games was strongly influenced by the interaction between the humans and their landscape. This is illustrated by the fact that sports equipment and tools for playing were largely made of materials provided by the surrounding biota. The objective of this article is to investigate various aspects of local knowledge about organisms used in the material culture of traditional Sámi sports and games. Which functions had the surrounding biota in the production of equipment used in traditional sports and games?

To map out and describe the use of the biota, we used qualitative methods and perspectives. Extensive literature research in libraries in Stockholm, Uppsala, Helsinki, and private collections yielded much new information about traditional sports and games among the Sámi. We particularly searched for information on the type of materials used to make toys and sports equipment. Records were obtained from a variety of primary and secondary sources. The traditional sports and games are mentioned in most older ethnographic descriptions of the Sámi, but the traditions have almost completely disappeared, so we cannot verify their accuracy by a modern comparison [12]. This study contributes data to the material culture of traditional Sámi sports and games from an ethnobiological perspective that is their associations with local knowledge of the biological environment [17]. The methods used here can best be described as cultural and historical, as we have mainly used texts, objects, and other materials to enrich the understanding of the past. Data about Sámi sports and games have been gathered from primarily ethnographic literature and travel narratives, but also material objects have been researched. The sources record centuries of Sámi indigenous knowledge and reflect the importance of sports and games for the Sámi people in the premodern time [7].

## Scientific framework

The ethnobiological perspective here referred to is the study of human dependency, knowledge, and activity context with the surrounding biota and landscape [17]. Anthropologist Claude Lévi-Strauss wrote about the *science du concrete* “science of the concrete,” typical in preindustrial, low-technology societies. Such societies show an exceptional familiarity with the biological environment; especially hunter-gatherers, pastoralists and peasants pay close attention to the biota [18].

**Fig. 7:**
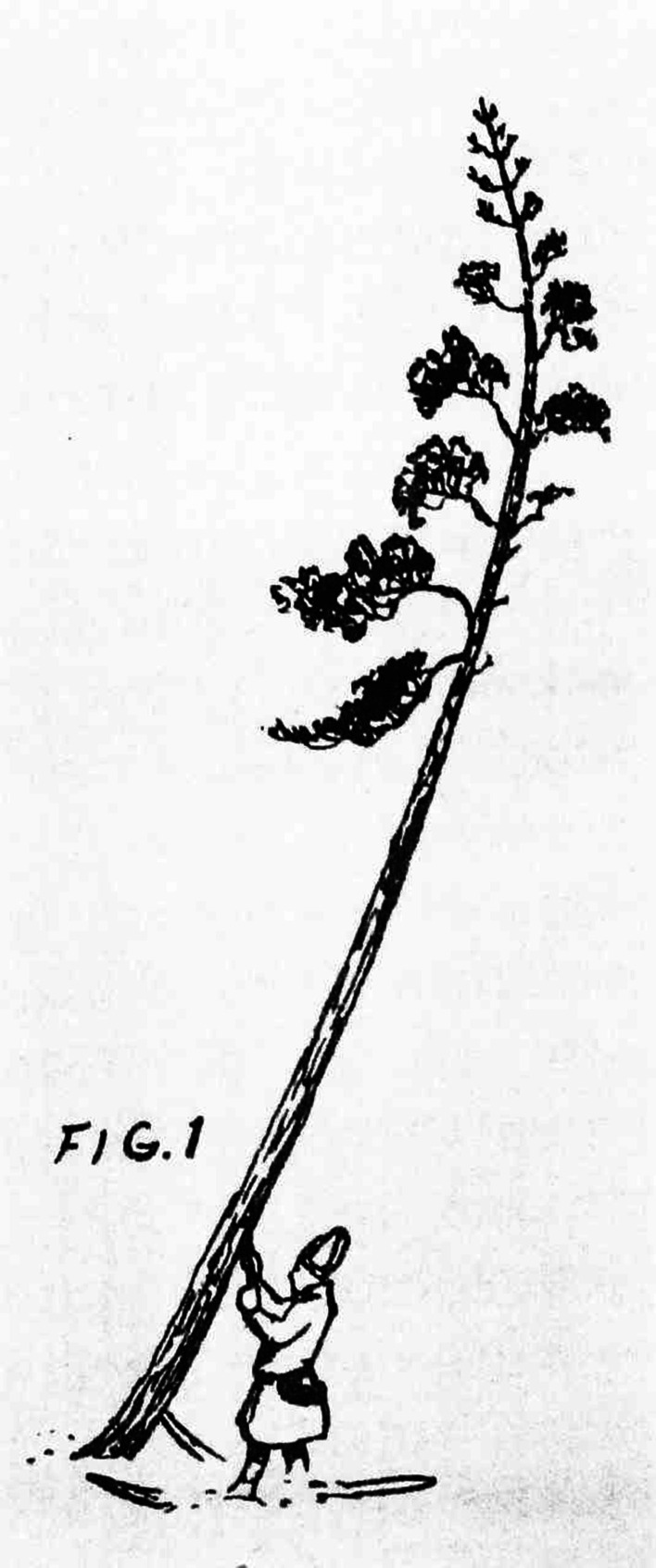
Sámi taking compression wood from a pine, *Pinus sylvestris* (*Samefolkets Egen Tidning*, 1928, no. 1)

In the case of the Sámi, part of whom were fishers and nomads, and another part settled crofters, such a “science of the concrete” of animals, vascular plants, lichens, bacteria, and other organisms, was a prerequisite for survival around and beyond the Arctic Circle in premodern times. Their knowledge of the qualities and usability of different kinds of wood, for instance, was large. The Sámi were aware of the usability of compression wood, which is formed on the lower side of a pine stem, and they used it for bows, skis, and sledges (Fig. 7). They also knew that the burr on a birch tree was useful for various handicrafts [19, 20]. For their survival as hunters, fishers, and reindeer herdsmen, the Sámi were forced to acquire an in-depth knowledge also of other species in the forests, mountains, and watercourses [21].

**Fig. 8:**
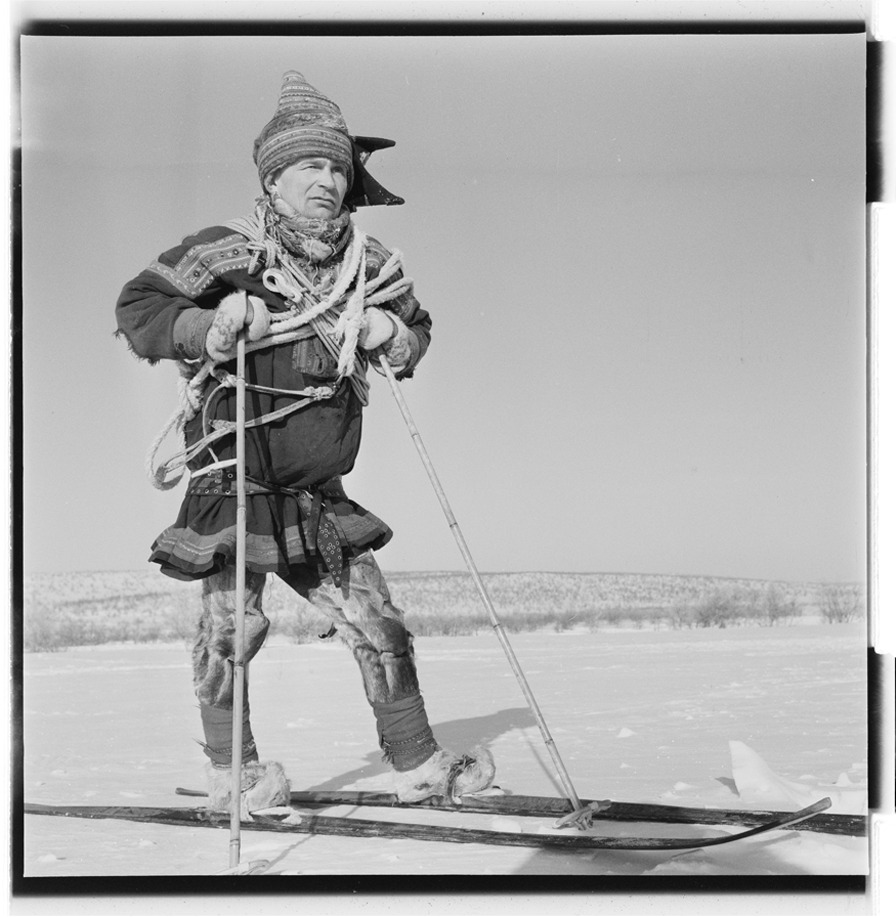
Norwegian Sámi reindeer herder on skis made of wood in 1962. Note the lasso (Photo Lasse Klaeboe. National Archives of Norway)

The landscape, in which the Sámi herdsmen, hunters and fishers operated, was not untouched by humans: It was an outcome of the work, knowledge, and zeal of the inhabitants for several generations. Humans manage their environment through domestication, hunting, and trapping, as well as through constantly striving to increase production in different ways. They do this by transforming the landscape, something that not only affects the biological diversity, but also their relationship with other species in the environment (Fig. 8). The landscape displays a vast natural variety in the far north of Europe (coasts, fjords, rivers, mountains, forests, tundra, lakes, etc.), and simultaneously it contains at any given moment a biological cultural heritage [22]. The abundant Sámi snow terminology, for example, reflects their detailed knowledge about the landscape, and specific and important aspects of it for humans living in this environment [23, 24]. Different biocultural domains have produced special Sámi terminologies, which should be studied further, since they provide insights into how various organisms and landscapes affect the several Sámi groups’ ways of thinking and lifestyle [25]. Such in-depth studies of terminology and concepts could provide a deeper ethnographic understanding of the relationship between human groups and other organisms in a specific ecosystem [17, 18, 20, 21].

Ethnobiologists study cultural heritage by focusing on the biocultural domains arising in the interaction between human and other species in the environment. The different Sámi groups mastered their landscape and possessed a great familiarity with both qualitative and quantitative aspects of other species, with whom they shared their habitat. They knew what could be used for feeding animals, food, medicine, utensils, fuel, hygiene items, dyes, poisons, and other necessities, essential for survival in the subarctic environment [26, 27]. Yet other organisms were sometimes seriously affected by human exploitation and existence in the same ecosystem: For instance, when the Sámi in early modern times enlarged their herds of semi-domesticated reindeer, wild reindeer, *Rangifer tarandus* (L.), was wiped out and vanished from the region [28].

## Results

Sports and games performed by the Sámi have been recorded in ethnographic literature since the seventeenth century until a century ago [9, 12, 29, 30]. In addition to sports and games requiring tools, here were also non-formal games and contests, which did not require any special instruments, such as walking on hands, standing on the head, or running [31]. Tree climbing provided a popular summer activity. Birches were used for jumping: Boys climbed into a tree, which bowed down because of their weight, and then they hopped off it. In winter, sleighs and ice-racing were popular. Lifting stones or other heavy objects and carrying them over a certain distance were also common games. In this article, however, we focus on the material culture and the production of tools for sports and games, and we present different biological and natural resources, such as birch polypore to make balls, reindeer antlers for lassoing contests, compression pine for skis, and pine bark painted with reindeer blood, which were used for playing cards [11, 12] (Table 1).

**Table 1:** Biological resources used for making sport and game equipment and toys

Species	Part		Used for	**Sources**							
**Mammals**											
Reindeer, *Rangifer tarandus* (L.)	Antler		Lasso loop Glue for making skis	[11, 38] [36]							
	Fur Leather Kneecap Inflated stomach Bone		Skis Lasso Toy reindeer Buoyance device Glue	[36] [38] [12] [9] [30, 36]							
Lemming, *Lemmus lemmus* (L.) Brown bear, Ursus arctos L **Birds**	Living animal; skin Fat		Toy Ski wax	[36] [30]							
Wild goose, *Anser* sp.	Sternum		Toy reindeer antler	[12]							
Ptarmigan, *Lagopus* sp*.* Whooper swan, *Cygnus cygnus* (L.), **Fish**	Crop Flight feather		Musical instrument Bird whistle	[12, 30, 36] [3]							
Pike, *Esox lucius* L	Vertebrae		Toy necklace Children’s games	[3, 11, 30, 36] [3]							
European perch, *Perca fluviatilis* L Unknown big fish **Shellfish** Great scallop, *Pecten maximus* (L.) Blue mussel, *Mytilus edulis* L **Fungi**	Fish scales Swim bladder Shell Shell		Glue for making skis Noise maker Toy animals Toy animals	[37, 48] [11, 30] [13] [13]							
Birch polypore, *Fomitopsis betulina* (Bull.) B.K.Cui, M.L.Han & Y.C.Dai	Fruit body		Ball	[6]							
**Vascular plants**											
Scots pine, *Pinus sylvestris* L-	Compression wood		Skis Bow	[11, 36] [49]							
Norwegian spruce, *Picea abies* (L.) H. Karst	Wood Roots Wood Wood		Bow, Crossbow Lasso Stilts Skis	[9] [36, 38] [12] [36]							
Juniper, *Juniperus communis* L Downy birch, *Betula pubescens* Ehrh	Branches Tree bark Wood		Bow, crossbow Toys Birch bark trumpet Skis Walking stick	[9] [12] [37] [12, 36] [31, 53, 54]							
			Ski pole	[9]							
Dwarf birch, *Betula nana* L Rowan, *Sorbus aucuparia* L Goat willow, *Salix caprea* L	Branches Branches Branches		Toys, fuel Flute Flute	[37] [30, 37] [11]							
Norwegian angelica, *Angelica archangelica* L	Stem		Flute Toy tobacco pipe	[10] [11]							
Mezereon, *Daphne mezereum* L Stag's-horn clubmoss, *Lycopodium clavatum* L Turnip, *Brassica rapa* L. *subsp. rapa* Common reed, *Phragmites australis* (Cav.) Trin. ex Steud	Fruits Branches Root Stem		Used in flute Wrath Ball Flute	[9, 11] [37] [12] [30]							

The preparation of tools and toys began usually with the birth of a child. Toys were prepared by the grandparents, or family members of the same gender as the baby. The cradle was made of wood, lined with reindeer skin, and decorated with shiny objects of silver or brass, and other ornaments for the baby to play with. The Sámi added earlier also small toys or objects into the cradle, or hung them in a bag at the foot of it, reflecting their material culture and good wishes for the future. This custom apparently changed over time, and it gradually disappeared during the nineteenth century. For boys, there would be miniature skis, boots, a spear, bow and arrows made of tin, and for girls the wings, feet and beak of a ptarmigan, which meant that the girl would become as clean, capable and quick as the bird (it turns white in winter) [12].

### Balls and ball games

The ball is one of the oldest and globally most widely distributed game and sports tool [6]. Before the emergence of factory-made balls, the Sámi put a lot of effort into making different kinds of balls, from simple snow balls to more advanced ones. They used, for instance, round stones, reindeer antlers, or even turnips (*Brassica rapa* L. ssp.* rapa*). The turnips the Inari fisher Sámi threw at each other over a pot with boiling water. Some Sámi groups also used birch slings with stones for competitions, and many played with skittles [12].

Travelogues and ethnographic literature from the seventeenth and eighteenth centuries provide detailed descriptions of ball games played by the Sámi with different kinds of balls. In 1673, Johannes Schefferus described several games, which were played with balls from materials from both animals and plants. One ball was “big as a fist, made of leather, and stuffed with hay” [32]. He described also a game with a bat and ball, and a stick-ball game. The latter was played on the snow crust in a defined area with two borderlines marked at the edges. Equipped with sticks and bats, the two teams aimed to drive the ball across the boundary line of the opponents. The game resembles largely shinty and hurling, ancient stick-and-ball games of Scotland and Ireland [6, 32].

**Fig. 9:**
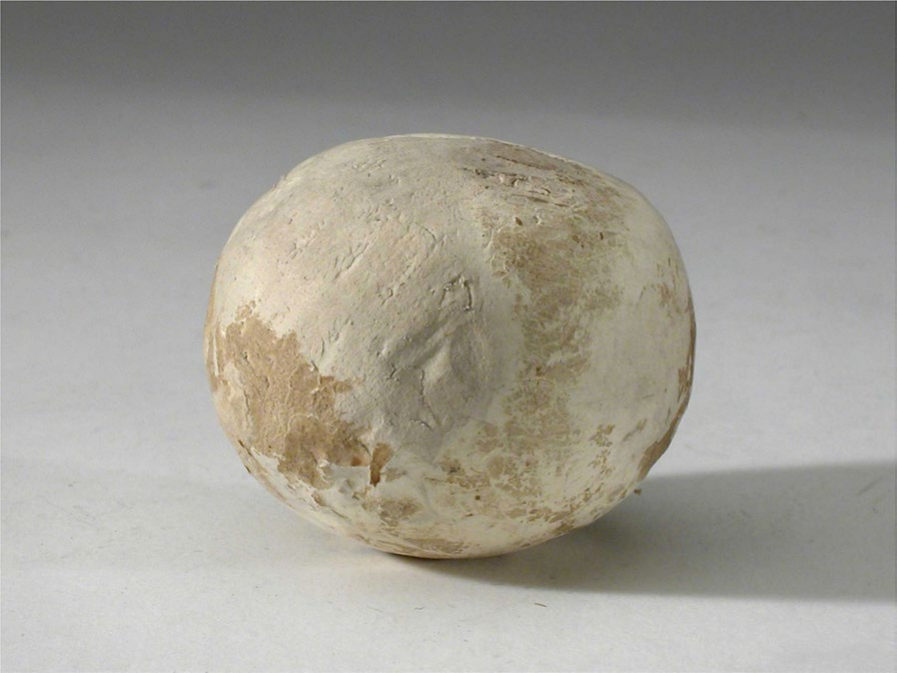
A ball made of birch conk from the late nineteenth century, made at Mattisudden Sámi handicraft school. Courtesy of The Nordic Museum, Stockholm

**Fig. 10:**
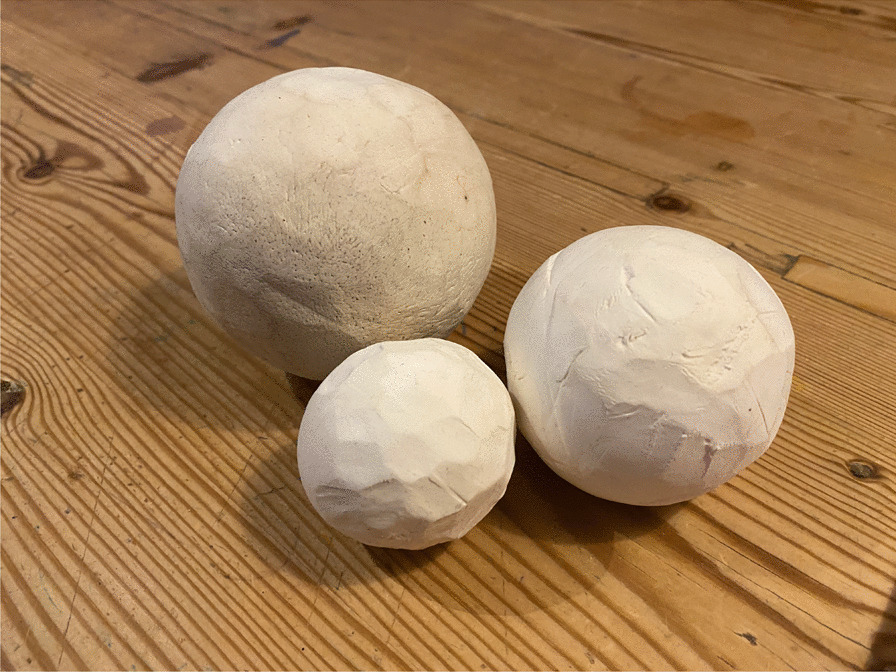
Newly made balls of birch conk. Photo Isak Lidström

The Italian explorer Guiseppe Acerbi described a game with a similar kind of ball in the late eighteenth century. The Sámi played a game “with a leather ball, stuffed hard” [33]. Another eighteenth-century source, by the Norwegian vicar and linguist Knud Leem, tells of a ball “covered with hide and stuffed with hair, straw, rags, or the like” [34]. The latter game was a widespread European base running game, known under different names such as “longball” in the Nordic countries, *lapta* (Russia), *Schlagball* (Germany), or *palant* (Poland) [35]. Another material, common in the manufacture of balls, was the bracket fungus *Fomitopsis betulina* (Bull.) B.K. Cui, M.L. Han & Y.C. Dai [6]. (Figs. 9, 10) The Inari Sámi in northern Finland boiled the fungi in ash lye to make them elastic [12]. Among the Sámi by the Lule River in Sweden in the early nineteenth century, balls were made of white bracket covered with chamois leather [6, 12, 36].

### Reindeer games

**Fig. 11:**
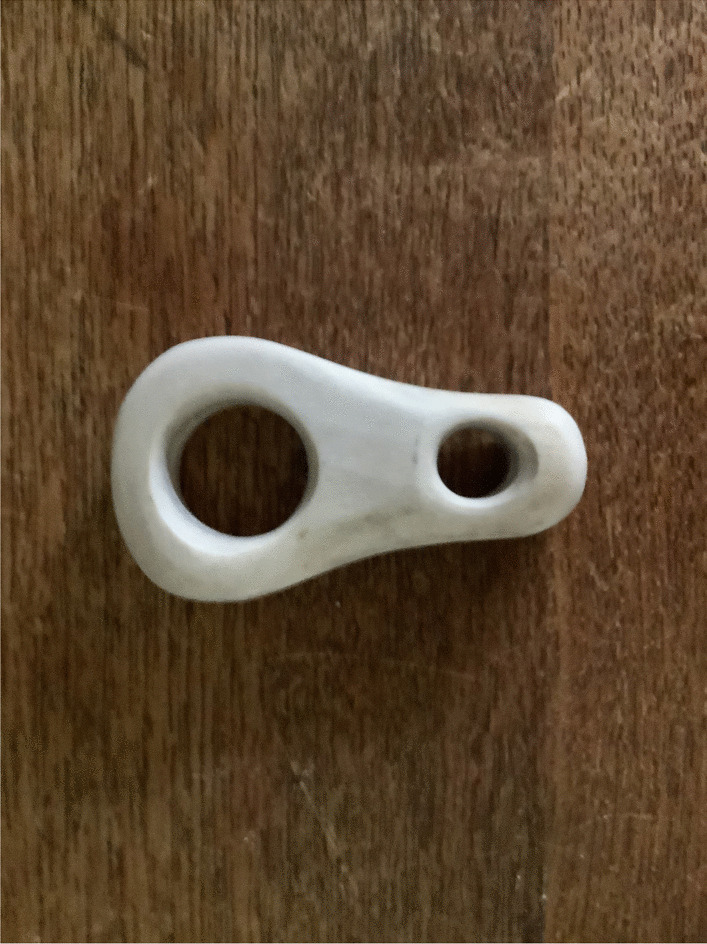
Lasso loop (Lule Sámi *sjuohpanrikkas*) with reindeer antler for lasso made in the 1980s by Sune Enoksson in Tärnaby, Västerbotten, Sweden (Photo Ingvar Svanberg)

Reindeer husbandry is strongly connected with several Sámi groups and cultures, and reindeer are to a high degree reflected also in sports and games (Fig. 11). When the famous naturalist Carl Linnaeus in 1732 traveled among the Sámi, he observed and described many leisure-time activities and games, too. He wrote in his diary that the children made reindeer antlers, for instance, of dwarf birch, *Betula nana* L. Pretending to be reindeer, they would fight one another [37]. An important skill for a reindeer herder was to catch the animal. Linnaeus noted that the children played reindeer games in a special paddock, set aside for these games in the settlement. One child would wear reindeer antlers (small for calves, middle for cows, and big ones for bulls), or use bushes, or their hands to mark that he or she was a reindeer. The others played humans, wolf, or dogs (the dogs would try to “bite” the “reindeer’s” legs). Both girls and boys played this game. Through playing, the children learned early the significance of size, color, and form of reindeer, terminology for antlers and other reindeer-related words, and skills like dividing a herd, catching the animals, keeping dogs or wolves at bay, etc. [11, 12]. A more challenging strength test among reindeer herders was to try to tear off the tendon located under the knee crease of a dead reindeer – far from all were successful [11, 12].

Children sometimes used the lasso as a simple swing, *skeälbbuo* (Ume Sámi), according to records from Arvidsjaur (Pite Sámi *Árvehávvre*) [9, 36]. Traditionally the lasso, *soehpenje* (South Sámí), was made of twisted reindeer leather, *garhtseke soehpenje*, hard twisted leather and tendons, or of twisted spruce roots, *veadtehke soehpenje* [38]. Handling a lasso is a professional skill, but also an arena for play, fun, and competition. A recurring motif within the ethnographic literature is Sámi children at play, one holding a reindeer antler above the head, and the others trying to catch the “reindeer” with a lasso. The lasso could also be thrown over bushes and stubs, coffee kettles, or even dogs, which were more real and moving targets. Inside the hut, a rope could be fastened to a bed, and the children played at driving a reindeer sleigh, training the necessary movements with the reins. Reindeer games of this kind reflected the life of the adults, and the children practiced different skills needed for animal herding [11, 12]. According to the nomad Sámi Anta Pirak, a lasso was often the youngest children’s first toy. At age eleven, a boy would receive a real lasso [12, 39].

**Fig. 12:**
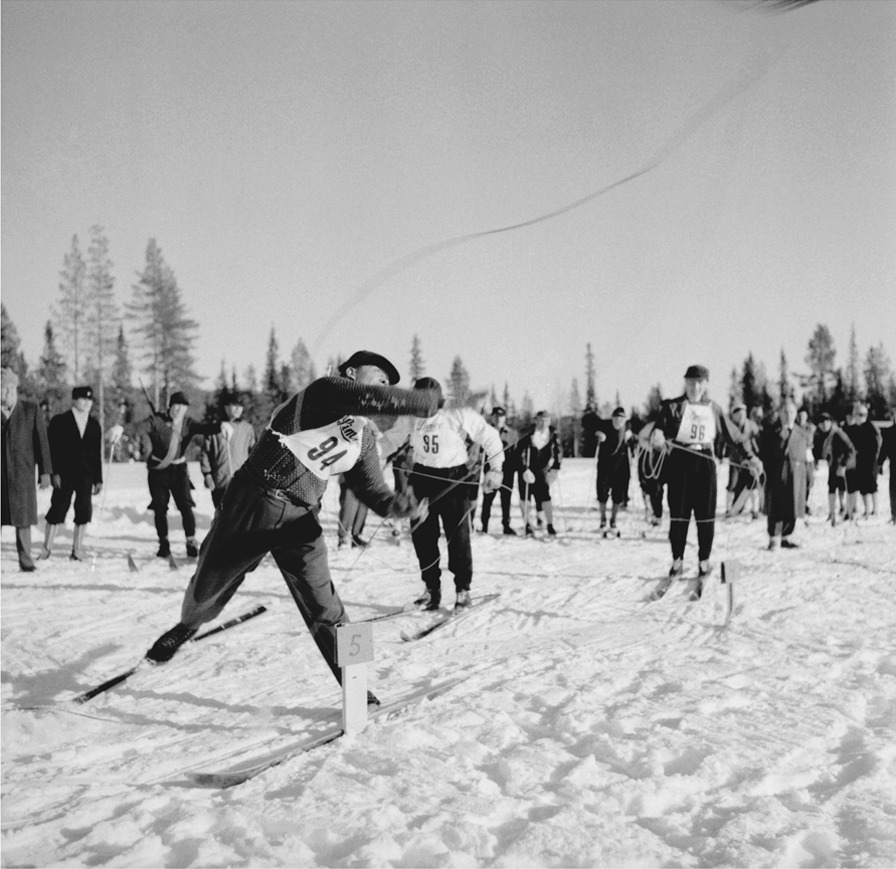
Lasso competition at the Sámi championship in Vilhelmina, Sweden, in 1950. (Photo: Åke Sörlin. © Vilhelmina Kommuns Fotoarkiv

In the Nordic countries, there is a separate Sámi sports movement dating back to 1948, the year when the first Sámi Championships were organized in Jokkmokk (Lule Sámi *Jåhkåmåhkke*) in Sweden [40]. Among the competitions, there were a reindeer herders’ relay race, a patrol competition in which three-man teams skied, threw lasso over reindeer antlers, and shot at targets in the shape of wolves. This was the emergence of lassoing as a modern sport, which is still performed during Sámi competitions [41]. During the process of sportification, lassoing as a computational activity has come to differ from lassoing as an occupational skill; a reindeer herder throws the lasso over a reindeer in motion, while the athlete throws at a stationary target [41] (Fig. 12).

The ethnobiological aspect of the sportification of lassoing is noteworthy. Considered a sport, standardized rules and equal conditions between the competitors are required [42]. The latter criteria are difficult to meet, however, since the target consists of reindeer antlers, which are all unique in appearance and shape. This has posed a dilemma for Sámi sportsmen. Over the last decades, voices have called for a change of rules, suggesting a replacement of reindeer antlers with a straight pole, but such proposals have consistently been rejected. The reindeer as a symbol of Sámi culture and Sámi identity has been considered more important than the equalization of competitive conditions. In 2012, a hybrid solution was proposed, with the introduction of standardized reindeer antlers made of plastic. A scholar at Luleå Technical University in Sweden was commissioned to construct such a target and completed the assignment by digitally scanning real reindeer antlers, from which a prototype was developed. The new targets were never introduced at the lassoing competitions, however, and the project of standardizing lasso targets ended at the prototype stage [43, 44].

### Skiing

The art of skiing is a winter sport, which traditionally has a strong connection with all Sámi groups [36, 45]. Children still learn skiing at an early age. The Sámi hunted and moved in the surrounding environment in winter on skis. They slid down mountain slopes for fun, but ski competitions were rare; instead, reindeer sleigh contests were popular [12]. Although it is impossible to determine who invented skis as a means of transport, or when this happened, “the vast majority of historical accounts make it clear that the Sámi were highly skilled skiers, and that the art of skiing made up an important part of their cultural identity,” the Sámi scholar May-Britt Öhman states [46]. The Sámi were not the only ones to use skis in the Nordic countries; according to the archaeologist Inger Zachrisson, the Sámi probably even manufactured skis for other people of the North. The majority of archaeological ski finds in Fennoscandia are of Sámi origin. The specialization in skiing can be traced back to the ninth century [47].

Linnaeus described in the eighteenth century the way of making skis among the Sámi groups he visited: they used compressed wood, usually called *tjur* or *tiör* (northern Swedish dialect words). By using glue made of the skin of perch, *Perca fluviatilis* L., the pieces of wood were fused together [36, 48]. The skis were manufactured by compressed wood from Scots pine, *Pinus sylvestris* L., and by downy birch, *Betula pubescens* Erhard, which was also used in the traditional Sámi bow [49].

The Sámi nomad Anta Pirak described in the 1930s how such skis were made: Birch was used for the outer part of the ski’s underside, and compressed pine provided the inner part. Only by combining the two, a ski would get the right bend. A ski containing compressed wood was supposed not wear out so quickly on the snow crust. In thawing weather, the snow would stuck onto the compressed wood, rather than on the birch wood [37].

An important written source discussing Sámi ski culture is the Latin-language comprehensive work *Historiae gentibus septentrionalibus* (“History of the Nordic Peoples”), written by the Swedish Catholic ecclesiastic and author Olaus Magnus, and published in 1555. In his account on the northern *Scricfinnia*, Olaus Magnus describes among others skis covered with reindeer skin; the skin made it easier to move through deep snow and to avoid dangers such as deep cliffs. The fur also prevented the skis from sliding backwards when going uphill [50].

Although this early account of skiing was presented as a means of transportation, Olaus Magnus also stressed that skis were used for non-utilitarian purposes and for competitive activities. The people “try their Skill, and to contend for mastery therein, as those who run Races to win the price” [50]. When modern ski sports emerged in Sweden in the late nineteenth century, Sámi skiers dominated in the most prestigious competitions [51].

### Fencing with walking sticks

**Fig. 13:**
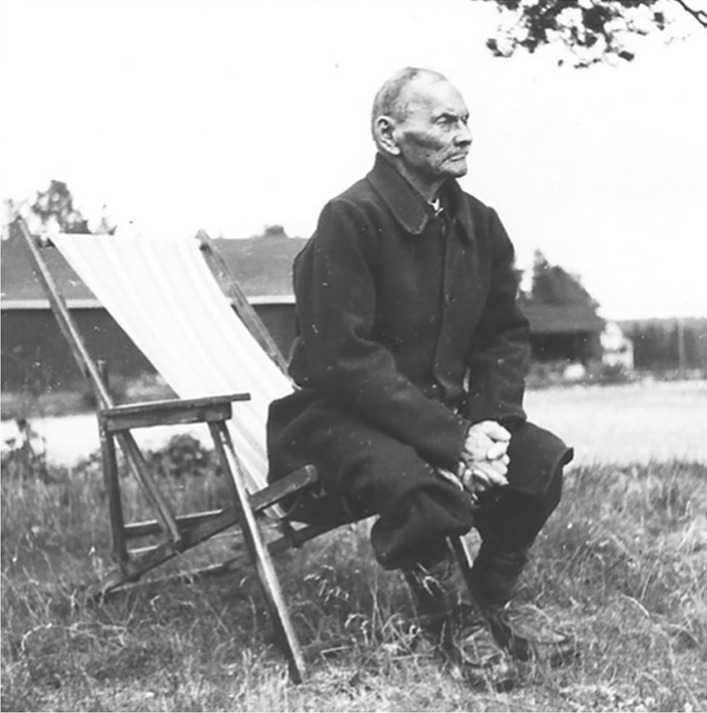
Per Larsson Gimmon (1858–1947) was known for his skills as a fencer using his walking stick. (Unknown photographer. Private archive of Carina Lasko)

If skis and poles were important means of transport during winter, the counterpart during summertime was the walking stick. The sticks were usually made of birch and filled a multitude of functions, for example as an aid when walking in difficult terrains or wading through streams. Sticks could be placed over the shoulder, functioning as a yoke to carry water buckets, or luggage [52]. The walking sticks were further used for competitions. The Sámi art of fencing with walking sticks is known from several historical records [36]. In the 1820s and 1830s, the Swedish priest and missionary Petrus Læstadius traveled in northern Sweden, and he mentioned in a record from 1831 that the Sámi used their long birch wood walking sticks for fencing with each other. The border between friendly competition and violence was blurred, and serious fighting occasionally characterized the art of fencing. One particular man, Anders Enarsson, was known under the name *Skalok*, which can be translated as “The Fighter” or “The Master of Fencing.” One of the combats he fought was fatal, resulting in the death of his competitor [31, 53]. Another Sámi known for his skills in fencing with a walking stick was Per Larsson Gimmon (1858–1947) who lived by the lake of Tjeggelvas (Lule Sámi *Tjieggelvas*), north of the village of Arjeplog (Pite Sámi *Árjapluovve*) (Fig. 13). He was known for his agile movements, and he fenced both standing, sitting, and lying down [54].

### Card and board games

In his famous book, *Lapponia,* published in 1673, Johannes Schefferus noted that the Sámi used to buy playing cards from visiting itinerant traders [32]. Among Coastal Sámi in Norway, it was common to make playing cards from the paper packaging of sugar-loafs [11]. The most conspicuous note is from a book published in 1747 and written by Pehr Högström. In his description of Sámi in Sweden, Högström noted that the playing cards were made of pine bark, which were painted with reindeer blood [55].

**Fig. 14:**
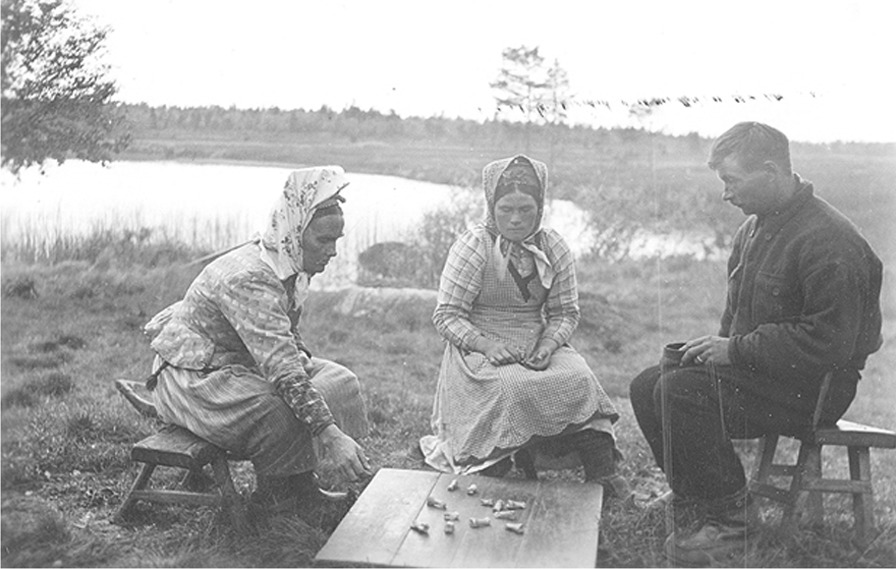
Skolt Sámi plays a traditional game, Petsamo, Finland, in 1935 (Photo Karl Nickul; The Finnish Heritage Agency, Helsinki, Finland)

Materials from reindeer were used in making board games (Skolt Sámi *sáhkuu;* South Sámi *daabloe;* Lule Sámi *dábllo*), which are known from the entire traditional Sápmi area (Fig. 14). The oldest written descriptions of such board games are from the eighteenth century, but the games were still played in the early twentieth century [36, 37, 56]. Not seldom the game pieces were made of reindeer hoofs, bones, coffee beans, or sticks with the bark intact for one player, and without for the other. The board was, for example, the back of a board used for scraping hides or baking, a reindeer skin, or a specially made board. The owner often engraved his mark on the side of the board. These games were usually local variations of games borrowed from neighboring peoples, and they were very popular [11, 12, 36, 56, 57].

## Discussion

In 1995, sports historian Henning Eichberg wrote that the “commercial market of body culture is expanding, threatening the traditional system of club sports.” In relation to this development, Eichberg also identified counter movements “redefining or re-practicing” the popular (Swedish *folklig*) “grassroots aspect of sports” [58].

Sports face today several challenges, due to increased globalization, universalism, and commercialization. The production of sports and games tools, clothes, and accessories is growing; fashions create a “need” to buy more and specialized items, and new sports or variations of sports and games are being developed and launched to attract more consumers. The modern industry of sports goods offers a vast range of highly developed products, which, however, draw on multiple natural resources for production. They leave a huge carbon footprint, being mostly manufactured in countries, where production facilities do not follow strict environmental rules, and cause various kinds of pollution. The consumption of sports equipment results at the consumer end in enormous amounts of litter, which cannot be reused or recycled, and they contain plastic and other materials contaminating and destroying ecological systems. Further, climate change stands out as a circumstance articulating the need of a new direction also for sports and games equipment and tools [59, 60].

**Fig. 15:**
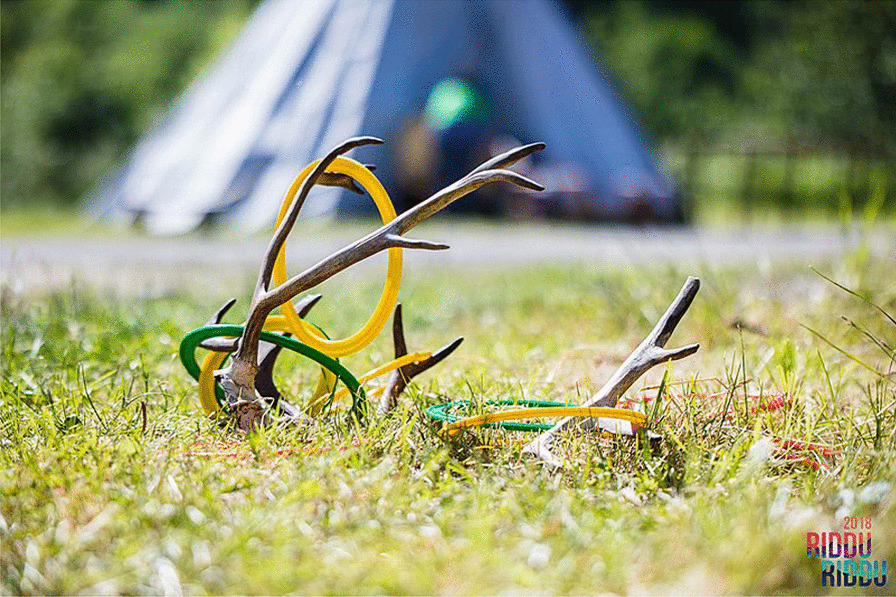
Reindeer antler used in a game at the *Riddu Riđđu* festival, Kåfjord, Norway (Photo Eirin Roseneng. Wikimedia Commons)

Considering environmental sustainability, the “grassroots aspect” is, to speak literally, more accentuated within sports today, compared with the context in which Eichberg wrote his article in the mid-1990s. The scholar Bente Ovedie Skogvang states that traditional Sámi sports, physical and outdoor activities at local festivals (such as the *Riddu Riđđu* festival in Kåfjord in Norway, and the annual winter festival in Jokkmokk, Sweden, *Jåhkåmåhke márnán*) gain an increased importance in the formation of a Sámi identity [40, 41, 61, 62] (Fig. 15). With an increased interest in traditional games and sports, where local natural and biological resources play a significant role, a potential development could be a fusion between sports and games on the one hand, and outdoor life and education (Swedish *friluftsliv*) on the other hand.

## Conclusion

A reintroduction of traditional games and materials from nature could provide an alternative concept, in which sports and games practices take place in the surrounding landscape, rather than in modern arenas with standardized dimensions, or in fitness clubs. Games of the past, compared with modern sports of today, show a crucial difference in the dependency between the practice and the surrounding biota. For instance, the shape and appearance of a ball depended on the natural resources available, and not on standardized formats and materials.

The Sámi culture of traditional games and sports was characterized by a great deal of creativity and innovative solutions in how the biota was used for play, education, and competitive purposes. The practice of games and sports was not only a physical activity; it was also a practice of interaction between human beings and their surrounding landscape, teaching children important skills and knowledge, keeping physically and mentally fit throughout the year, training different kinds of skills both necessary for survival and considered useful in the group, socializing, and having fun together. By reconnecting to the “grassroots aspects” of traditional games and sports, a revitalization could perhaps put Homo Ludens (“the playing human”) back into the landscape, use less non-reusable materials and resources, and provide a potential new direction for modern sports, shifting from globalization to environmentalization. An increasing environmentalization would let the local environmental context define the content, meaning and structure of sports, and simultaneously blur the border between sports and outdoor life, and enrich both.

## Data Availability

All data generated or analyzed during this study are included in this published article.
